# Herd immunity: challenges and the way forward in Korea

**DOI:** 10.4178/epih.e2021054

**Published:** 2021-08-18

**Authors:** Jiyoung Oh, Sohyun Kim, Boyeong Ryu, Minjoung Shin, Bryan Inho Kim

**Affiliations:** Division of Risk Assessment, Korea Disease Control and Prevention Agency, Cheongju, Korea

**Keywords:** SARS-CoV-2, COVID-19, Herd immunity, Transmission

## Abstract

Vaccination is considered to be the most effective measure for preventing the spread of coronavirus disease 2019 (COVID-19). Many countries, including of Korea, are focusing on achieving herd immunity with the goal of reaching a vaccination rate of 70-80%. However, achieving herd immunity does not mean eradicating COVID-19, and the following challenges can occur in the process of achieving herd immunity. First, as the vaccination rate is likely to slow down over time, it is necessary to promote the benefits of vaccination through risk communication strategies and provide incentives for those who have been vaccinated. Second, a booster dose may be required depending on future studies on vaccine-induced immunity. Third, since variants capable of evading immunity and with higher transmissibility can emerge, rapid contract tracing and regular community genomic surveillance could help mitigate the impact of new variants. When the impact of COVID-19 is controlled to the level of seasonal influenza, the current public health measures that have been strictly imposed on society since the beginning of the pandemic will no longer be needed. The overall response strategy to COVID-19 will need to change accordingly, based on evaluations of the level of population immunity. These changes will include more efficient and targeted contact tracing and eased quarantine measures for vaccinated close contacts and travelers. Mask wearing and a minimum of social distancing will still be required in the journey towards the end of the pandemic. The COVID-19 pandemic will end, but the virus will not disappear.

## INTRODUCTION

Since the World Health Organization declared the coronavirus disease 2019 (COVID-19) a Public Health Emergency of International Concern on January 30, 2020 [1], Korea has been recognized as one of the countries with a successful response to COVID-19 [2,3]. The Korea’s main interventions included rapid implementation of massive diagnostic testing, extensive contract tracing, transparent information sharing through daily media briefings, strict quarantine measures for travelers and contacts, and implementation of social distancing in collaboration with the general public. Although these measures have proven to be effective in controlling the spread of the virus, they have incurred an immense societal cost, which affects the sustainability of the response efforts.

## VACCINATION IS THE MOST EFFECTIVE WAY TO CONTROL COVID-19

At the beginning of the pandemic, with limited information about the novel coronavirus, different countries introduced different strategies to mitigate the spread of COVID-19. Sweden, supported by decision-makers and some scientists, stood out as a country with a unique approach to controlling COVID-19 by introducing a “focused protection” strategy. This strategy allowed less susceptible young people to live normal lives to obtain natural immunity through infection, while applying more protective and aggressive measures to protect highly susceptible groups until a vaccine became available to fill the gap to reach the herd immunity threshold [4]. However, the Swedish attempt to build natural herd immunity turned out to be unfeasible, and such a strategy is now considered somewhat irresponsible [5], as Sweden has experienced a heavy disease burden with a 4-10 times higher case fatality rate than its neighboring countries [6]. In contrast, a few countries, such as Vietnam and Taiwan, were successful at controlling COVID-19 through complete closure of their national borders [7,8]. However, the recent surge in new cases shows that the existing measures without vaccination have clear limitations in keeping COVID-19 under control. During more than a year of social distancing, lockdowns, and a combination of various restrictions, many countries have experienced multiple waves of the COVID-19 pandemic, and vaccination is now one of the most important key factors to reduce the impact and the burden of this novel virus.

## HERD IMMUNITY DOES NOT INDICATE THAT COVID-19 WILL BE ERADICATED

It now appears that the most effective way to control COVID-19 is through high vaccination coverage, which is commonly referred to as a key to herd immunity. Herd immunity can be achieved when a sufficiently large proportion of the population is immune, either through vaccination or immunity developed through natural infection [9]. It is widely assumed that herd immunity will enter into effect when the proportion of the population with COVID-19 immunity exceeds the herd immunity threshold, which is generally predicted to be 70-80% [10]. However, achieving population immunity is not a simple matter because many factors in addition to vaccination coverage need to be considered, including the population structure and density, as well as uncertain factors such as duration of immunity and vaccine effectiveness. Furthermore, the vaccines are currently approved for only specific age groups, and it will take additional time until vaccination is available for those who are younger than 15 years of age due to safety issues. Furthermore, even with a successful vaccination campaign, achieving herd immunity will not lead to the eradication of COVID-19. According to a poll conducted by Nature, in which more than 100 scientists working on COVID-19 participated, 89% of respondents answered that severe acute respiratory syndrome coronavirus 2 (SARS-CoV-2) would be either very likely or likely to become an endemic virus. Several other experts also anticipate that SARS-CoV-2 will become endemic, which points to the need for a longterm strategy to control COVID-19 [11].

## ACHIEVING HERD IMMUNITY: CHALLENGES AND THE WAY FORWARD

Globally, the first COVID-19 vaccine was approved and rolled out in December 2020, and the Korean government began vaccination on February 26, 2021, with the goal of achieving 70% coverage of the population with at least 1 vaccine dose by September 2021 and achieving herd immunity by November 2021. The Korean government began the campaign by inoculating healthcare workers first, followed by residents in nursing homes and longterm care facilities and frontline response workers. Priority was also given to those aged 60 years and older and those at high-risk of contracting COVID-19. Since the beginning of the vaccination campaign, there has been a significant reduction in the mortality rate among older adults. It is expected that the overall mortality will gradually decrease to the level of seasonal flu, which is about 0.05-0.1%.

In order to successfully vaccinate a sufficient number of the population, evidence-based approaches in terms of prioritizing high-risk groups and effective risk communication strategies for people with vaccine hesitancy are crucial elements of national strategies, as the speed of vaccination uptake can be affected by a number of challenges. Israel has reported the highest vaccination coverage with the fastest vaccine roll-out worldwide, with 63% of its population vaccinated with the first dose as of June 15, 2021 [12], following a vaccination campaign that started on December 19, 2020. The COVID-19 incidence and mortality rates in Israel decreased markedly as the vaccination coverage increased. The Israeli government highlighted that Israel was about to return to “normal life.” However, the speed of the vaccination roll-out slowed down and the curve of the vaccination rate started to flatten after March 22, 2021, when 60% of the population had received 1 dose of the COVID-19 vaccine; the vaccination rate subsequently reached 63% on June 15, 2021 [12]. Similar patterns have been observed in the United States and other countries, with vaccination coverage of 40-50% [12]. The stagnant pace of vaccination in these countries could be attributed to vaccine hesitancy, misinformation about vaccine safety, and a lack of awareness about the true risk [13]. Incentives for vaccination have been introduced in many countries, and collective efforts have been made to motivate millions of people to get vaccinated. The United States Centers for Disease Control and Prevention released guidance on May 16, 2021, advocating that fully vaccinated people should resume their normal activities without wearing a mask. In addition, many states started offering a variety of rewards, including scholarships, free passes to state parks, gift cards, and lotteries as incentives to those who choose to be vaccinated. The Korean government also planned to ease restrictions on gatherings for fully vaccinated people and to offer various incentives, while emphasizing the importance of following preventive measures The time taken to achieve 70% or more vaccination coverage in the Korea is highly dependent on the degree of success of the Korean authorities in taking sequential steps to ease restrictions and in convincing people who are either hesitant or marginalized to take part in the national vaccination strategy. Even after reaching the targeted rate of vaccination, some groups will not be covered by vaccination—including children under the age of 15. Although this age group accounts for 13% of the total population in the Korea, vaccines are not yet available for them. A thorough risk-and-benefit analysis should be conducted for this age group, as they can play a major role in spreading the virus after mass vaccination in other age groups.

Inherent to the nature of infectious diseases, waning vaccine-induced immunity has been a concern because much remains unknown regarding the duration of immunity. The consistency of immunity is an important factor in calculating the level of vaccination coverage required to maintain herd immunity in real-world situations [14]. One of the critical aspects of herd immunity is the duration of immunity after vaccination [15]. According to a study on individuals with natural SARS-CoV-2 infection, neutralizing antibodies were detected for 3 months to 6 months [15,16] and have been reported for as long as 8 months [17]. Memory B cells, which last longer than neutralizing antibodies, increased until 150 days after infection, and were still present 8 months after infection [18]. In vaccinated individuals, the antibody response has been shown to persist at least 6 months after the second dose of the mRNA vaccine [19]. The question of how long immune protection lasts will be answered as the country makes progress in the nationwide vaccination program. It is likely that the national plans for vaccination for herd immunity might need to include a plan for booster shots based on the available evidence regarding the duration of immunity through vaccination.

The emergence of multiple SARS-CoV-2 variants is another challenge to achieving herd immunity. Multiple SARS-CoV-2 variants have been identified worldwide, starting with the Alpha variant, which was designated as a variant of concern on December 14, 2020. Many studies have reported that the majority of vaccines are effective against variants. The estimated effectiveness of the BNT162b2 (Pfizer-BioNTech, Philadelphia, PA, USA) vaccine 14 days after the second dose is 89.5% and 75.0% with the Alpha and Beta variants, respectively [20]. Another study showed that the effectiveness of 2 doses of the ChAdOx1 nCoV-19 (Oxford-AstraZeneca) vaccine was 74.5% with the Alpha variant and 67.0% with the Delta variant [21]. However, SARS-CoV-2 continues to mutate as it spreads globally, and these high-levels of vaccine effectiveness may not last long. As seen in the United Kingdom, which is currently dominated by Delta variant-driven cases, new variants can rapidly replace the previous dominant variant. The European Centre for Disease Prevention and Control announced that if a new variant is 50% more transmissible than the predominant viral strain, it will replace the predominant strain within 74 days [22]. Many of the major variants exhibit enhanced transmissibility due to a greater binding affinity to the receptor-binding domain to the angiotensin-converting enzyme 2 receptor than the wild type of SARS-CoV-2. This characteristic raises several concerns, as it affects the severity of the disease, the risk of reinfection, and vaccine effectiveness [23]. The emergence of a variant that has an increased ability to escape vaccine-induced immunity and the immune response acquired by natural infection may be unavoidable; however, rapid contact tracing and real-time genomic surveillance could help mitigate the impact of new variants. Nonetheless, real-time genomic surveillance and subsequent rapid contact tracing could reduce the impact of new variants.

## CONCLUSION

Herd immunity will be achieved only through herculean efforts by all parties involved in the national vaccination campaign. However, even after the Korea reaches the vaccination coverage target of 70% or more, the risk of sporadic outbreaks will remain high due to the importation of the virus from other countries until the supply of vaccines is adequate and administered globally. COVID-19 is likely to persist as a major respiratory pathogen, such as influenza A (H1N1pdm09). When the impact of COVID-19 is controlled to the level of seasonal influenza, the current public health measures that have been strictly imposed on society since the beginning of the pandemic will no longer be needed. The overall response strategy to COVID-19 will need to change accordingly, based on evaluations of the level of population immunity. These changes will include more efficient and targeted contact tracing and eased quarantine measures for vaccinated close contacts and travelers. Wearing facial masks and a minimum of social distancing will still be needed in the journey towards the end of the pandemic. The COVID-19 pandemic will end, but the virus will not disappear.

### Ethics statement

Not applicable as the manuscript did not involve any experimentation.
